# Evolution of Disease Modifying Therapy Benefits and Risks: An Argument for De-escalation as a Treatment Paradigm for Patients With Multiple Sclerosis

**DOI:** 10.3389/fneur.2021.799138

**Published:** 2022-01-25

**Authors:** Brandi L. Vollmer, Andrew B. Wolf, Stefan Sillau, John R. Corboy, Enrique Alvarez

**Affiliations:** Department of Neurology and Rocky Mountain MS Center, University of Colorado School of Medicine, Aurora, CO, United States

**Keywords:** discontinuation, multiple sclerosis, disease modifying therapy, infection, relapse, high efficacy, de-escalation, escalation

## Abstract

**Background:**

Strategies for sequencing disease modifying therapies (DMTs) in multiple sclerosis (MS) patients include escalation, high efficacy early, induction, and de-escalation.

**Objective:**

To provide a perspective on de-escalation, which aims to match the ratio of DMT benefit/risk in aging patients.

**Methods:**

We reanalyzed data from a retrospective, real-world cohort of MS patients to model disease activity for oral (dimethyl fumarate and fingolimod) and higher efficacy infusible (natalizumab and rituximab) DMTs by age. For patients with relapsing MS, we conducted a controlled, stratified analysis examining odds of disease activity for oral vs. infusible DMTs in patients <45 or ≥45 years. We reviewed the literature to identify DMT risks and predictors of safe discontinuation.

**Results:**

Younger patients had lower probability of disease activity on infusible vs. oral DMTs. There was no statistical difference after age 54.2 years. When dichotomized, patients <45 years on oral DMTs had greater odds of disease activity compared to patients on infusible DMTs, while among those ≥45 years, there was no difference. Literature review noted that adverse events increase with aging, notably infections in patients with higher disability and longer DMT duration. Additionally, we identified factors predictive of disease reactivation including age, clinical stability, and MRI activity.

**Conclusion:**

In a real-world cohort of relapsing MS patients, high efficacy DMTs had less benefit with aging but were associated with increased risks. This cohort helps overcome some limitations of trials where older patients were excluded. To better balance benefits/risks, we propose a DMT de-escalation approach for aging MS patients.

## Introduction

Multiple sclerosis (MS) is an inflammatory disease with associated neurodegeneration affecting the central nervous system (CNS) (1). Over 20 disease modifying therapies (DMTs) are now available that, in both clinical trials and real-world studies, reduce measures of disease activity including new relapses, MRI lesions, and accumulated disability (2). Given differences in efficacy, tolerability, cost, and safety, selection of DMT requires extensive shared decision making between clinicians and patients. These decisions are complicated and should be re-evaluated periodically as relapse and MRI disease activity generally decrease with patient age and duration of disease, likely reflecting a clinical measure of immunosenescence (3). Additionally, the risk of infections and, to a lesser extent, other adverse events frequently increase with age (4–7). Given the number of DMTs and variability of disease activity over the course of a lifetime, there is currently little consensus on the best approaches to treatment.

Here, we will explore what to do later in the treatment of patients with MS and provide our perspective to consider a de-escalation approach. We evaluate the effect of age on disease activity from a recent publication comparing rituximab to natalizumab, fingolimod, and dimethyl fumarate in a real-world setting of 1,246 patients (8). We apply lessons learned from the risk of rituximab in the real-world setting of 1,000 patients and other literature to better inform our perspective on de-escalation of therapy in the treatment of patients with MS (9). Additionally, we review the literature to identify factors associated with disease activity in DMT discontinuation studies to inform a perspective on how to use de-escalation as a treatment strategy.

## Methods

Our analysis utilized data collected for a prior retrospective observational study including participants who had an MS diagnosis; initiated rituximab, natalizumab, fingolimod, or dimethyl fumarate at the Rocky Mountain MS Center at the University of Colorado between January 2010 and October 2013; and, for natalizumab patients only, had a negative JCV serology test at baseline. Detailed methodology and study sample characteristics have previously been reported (8).

We dichotomized patients into two exposure groups defined as either receiving oral DMT or infusible DMT. Our binomial outcome was a composite effectiveness measure defined as the patient experiencing either a clinical relapse, a contrasting enhancing lesion, and/or a new T2 lesion on follow-up MRI within 2 years of drug initiation and while on treatment. The data was then modeled with generalized additive models for our binomial outcome, and penalized cubic regression smoothing splines for the effect of age for the entire cohort and separately by type of DMT (oral or infusible). Using this modeling approach, the age for which 95% confidence intervals overlap indicating no significant difference between groups was identified.

Additionally, for patients with relapsing forms of MS, we conducted a stratified analysis examining odds of disease activity for those on oral vs. infusible DMTs among patients <45 or ≥45 years of age. For this subgroup analysis, three models were used, including simple logistic regression, adjusted logistic regression, and logistic regression on sample group 1:1 nearest neighbor matched by propensity scores (PS) with replacement additionally controlling for covariates. Adjustment methods controlled for age, disease duration, sex, contrast enhancement on baseline MRI, and baseline disease burden (mild, moderate, severe, missing). As a sensitivity analysis, we also examined the outcome of clinical relapse individually using simple logistic regression and adjusted logistic regression.

## Results

**Figure 1:**
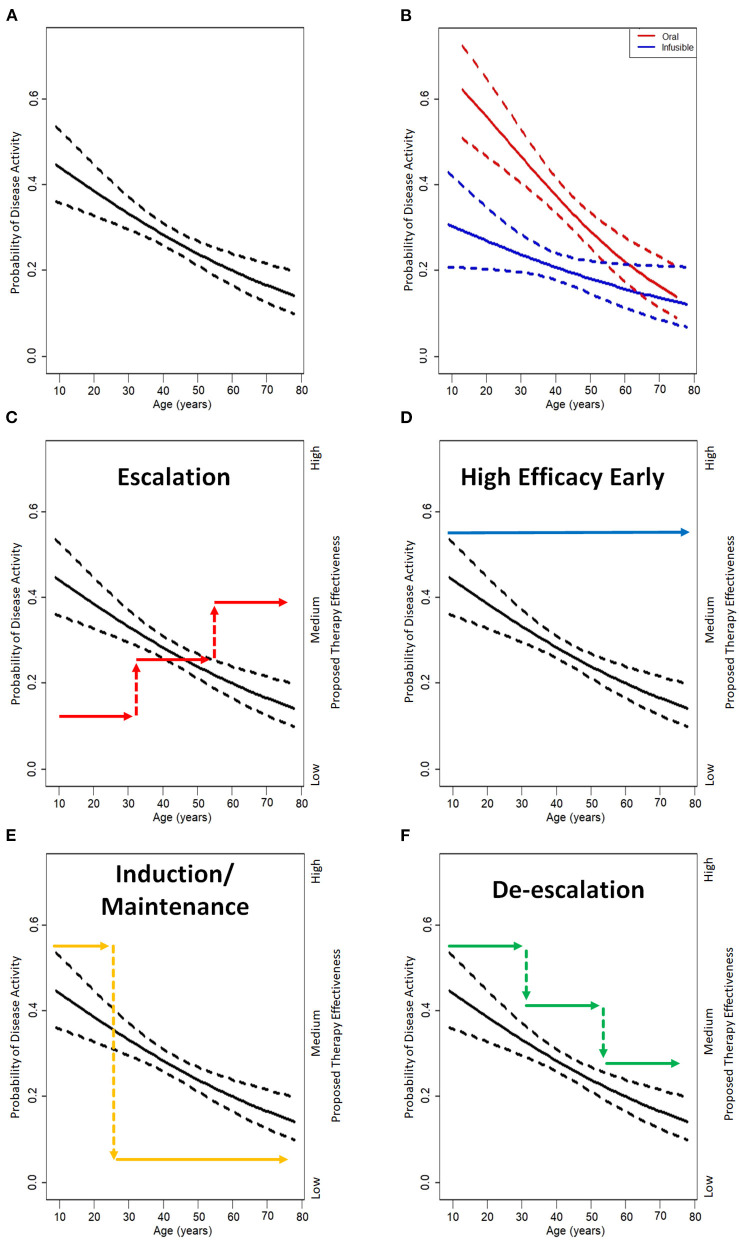
The probability of disease activity decreases over the lifetime of a patient with MS. **(A)** Probability of disease activity (clinical relapse, new T2 lesions, or enhancing lesions) observed in real-world study of 1,246 patients with MS with 95% confidence interval shown in dashed lines. **(B)** The probably of disease activity is higher in patients on oral (red; dimethyl fumarate and fingolimod) disease mofifying therapies (DMTs) than on infusible (blue; natalizumab and rituximab) DMTs. This probability is higher in younger patients and becomes non-significant by 54.2 years of age where the confidence intervals overlap. **(C)** Given the variable probability of disease activity, it is possible to observe that an escalation treatment approach under-treats early and over-treats as patients age resulting in possibly taking on higher risks but receiving little additional benefit from higher efficacy therapies. **(D)** High efficacy therapy early in the disease course matches the higher probability of disease activity early but can over-treat later in life. **(E)** Induction is often enough to sustain good efficacy early but some patients may breakthrough over time resulting in the need of retreatment or a maintenance therapy. **(F)** A de-escalation approach matches disease activity over a lifetime best, but will benefit from the use of better biomarkers to more rationally prompt changes in DMT. Shared decision making remains a crucial component of deciding on treatment approaches.

Our study included a total of 1,246 participants composed of 613 patients on oral DMTs (271 fingolimod, 342 dimethyl fumarate) and 633 patients on infusible DMTs (182 rituximab, 451 natalizumab). Figures 1A,B demonstrate the probability of experiencing disease activity within 2 years of drug initiation by age at time of drug initiation for the entire cohort and separated by type of therapy, respectively. When examining the probability of disease activity by type of therapy, there is a statistically significant difference between oral and infusible DMTs up until the age of 54.2, when confidence intervals begin to overlap.

Stratified analyses of patients with relapsing forms of MS included a total of 625 (276 oral, 349 infusible) patients <45 years of age and 379 (233 oral, 146 infusible) patients ≥45 years of age. Baseline characteristics are listed in Table 1. Among those <45 years of age, patients on oral DMTs had significantly greater odds of disease activity compared to patients on infusible DMTs [unadjusted odds ratio (OR), 2.67 (95% confidence interval: 1.89, 3.75), *p* < 0.001; adjusted OR, 2.89 (2.02, 4.13), *p* < 0.001; PS 1:1 nearest neighbor matching and controlling for covariates OR, 2.18 (1.34, 3.53), *p* = 0.002]. Among those ≥45 years of age, patients on oral DMTs had no significant difference in odds of disease activity compared to patients on infusible DMTs [unadjusted OR, 1.60 (0.96, 2.64), *p* = 0.069; adjusted OR, 1.65 (0.99, 2.76), *p* = 0.053; PS 1:1 nearest neighbor matching and controlling for covariates OR, 1.16 (0.59, 2.27), *p* = 0.675] (Supplementary Table 1). When examining clinical relapses individually, results were consistent. Among those <45 years of age, patients on oral therapies had significantly greater odds of relapse than patents on infusible DMTs [unadjusted OR, 2.82 (1.64, 4.83), *p* < 0.001; adjusted OR, 2.93 (1.71, 5.14), *p* < 0.001]. There was no significant difference in clinical relapses between oral and infusible among those ≥45 years of age [unadjusted OR, 1.27 (0.56, 2.92), *p* = 0.567; adjusted OR, 1.49 (0.67, 3.32), *p* = 0.328].

**Table 1:** Baseline characteristics for oral vs. infusible in patients with relapsing forms of MS.

	**Oral (*****n*** **= 1509)**		**Infusible (*****n*** **= 495)**		***p*-Values**
	***n* or mean**	**% or SD**	***n* or mean**	**% or SD**	
**Disease duration (years, SD)**	10.1	6.5	10.7	6.9	0.215
**Age (years, SD)**	42.5	11.6	38.6	11.5	<0.001
**Gender—Female**	370	72.7%	378	76.4%	0.182
Previous DMT^*^					<0.001
Interferons	74	14.5%	84	17.0%	
Glatiramer acetate	137	26.9%	150	30.3%	
Natalizumab	145	28.5%	60	12.1%	
Rituximab	6	1.2%	0	0.0%	
Fingolimod	17	3.3%	23	4.7%	
Dimethyl fumarate	1	0.2%	2	0.4%	
None	121	23.8%	165	33.3%	
Other	8	1.6%	11	2.1%	
**Contrast enhancement on baseline MRI**	90	20.4%	143	34.1%	<0.001
**Disease burden on baseline MRI**					0.629
Mild	232	45.6%	208	42.0%	
Moderate	141	27.7%	152	30.7%	
Severe	53	10.4%	49	9.9%	
Missing	83	16.3%	86	17.4%	

*DMT, disease modifying therapy; SD, standard deviation; n, sample size*.

* *Within 6 months prior to starting study drug*.

## Discussion

### Treatment Strategies

Studies of treatment strategies have primarily focused on choice of DMT early in the disease course, and switching DMT, typically due to intolerance or perceived loss of effectiveness. How to best sequence DMTs and for how long to treat patients with a DMT remain fundamental questions for which there is currently limited evidence. Strategies for sequencing include an escalation approach in which less potent DMTs (e.g., glatiramer acetate, beta-interferons, teriflunomide, S1P receptor modulators, or fumarates) are used first, with transition to more potent therapies if there is evidence of inadequacy (Figure 1C). An additional strategy is early use of high efficacy DMTs (e.g., B-cell depletion, natalizumab; Figure 1D).

There is increasing evidence that starting with high efficacy DMTs early in the disease course can result in better long-term outcomes compared to the escalation approach (10–12). This concept of treatment escalation is common in medicine. Proponents of an escalation strategy in MS argue that disease activity can be detected early enough for treatment to be effectively escalated and that any damage caused by this breakthrough disease activity is offset by the lower risks of these less potent DMTs. In neurological conditions such as MS, however, the concern is that this inflammation results in permanent damage to the CNS. In addition, current techniques to evaluate disease activity (including markers of progressive disease) are inadequate, and there is likely “silent” worsening separate from overt relapses that may be amenable to intervention and better treated with more effective DMT. Two trials are underway to further evaluate escalation and early high efficacy treatment—Effectiveness of earLy Intensive Vs. Escalation approaches for the Treatment of Relapsing-remitting Multiple Sclerosis (DELIVER-MS, NCT03535298) and Traditional Vs. Early Aggressive Therapy for Multiple Sclerosis Trial (TREAT-MS, NCT03500328). The issue of duration of use for either escalation or early high efficacy therapy is not addressed in these relatively short studies.

A third, less common, approach is use of induction therapies (Figure 1E), such as with alemtuzumab and cladribine. These medications are used infrequently, possibly because they are associated with higher perceived risks of infections, cancer, and/or autoimmunity. Autologous hematopoietic stem cell transplant has been used in small numbers of younger patients with highly active disease. All these approaches offer the possibility of immunosuppression that may be long-lasting or even permanent, but patients treated with induction approaches can go on to have disease activity long term which may require retreatment or a maintenance therapy. Thus, durability of these approaches, and duration of use of secondary therapies after their use remains unclear.

### Variable Therapy Effectiveness Over a Lifetime

Multiple sclerosis (MS) has inflammatory and degenerative components and, while the mechanisms and timing of transition to progressive disease course are often uncertain, aging plays a key role in both the accumulation of disability and the relative contribution from active inflammation (13). As all FDA-approved MS DMTs are immunosuppressive or immunomodulatory, this has led to assessment of therapies via phase III trials primarily in the population <55 years, when MS is typically most active. While there are substantial individual differences in disease activity across patients, DMTs will generally be most valuable early in the disease course and in younger patients. However, MS is most prevalent in patients in their late 50s, and many of these patients continue DMT (14, 15). Cohort studies indicate that relapse rates are age and time-dependent, with time measured from diagnosis; one study encompassing 2,477 patients over 20 years mean follow-up, relapse rate was proposed to decline by 17% every 5 years with this decline accelerating with age (16). This clinical data is supported by pathology data demonstrating that the rates of active plaques (correlating to enhancing lesions on MRI) decline with age and disease duration (17). Multiple phase III trials of DMTs currently approved for MS have demonstrated a higher efficacy in subgroup analyses of younger patients (18–22). This is supported by a modeling study of >28,000 MS patients treated with 13 types of DMT (ranging from interferons to ocrelizumab and siponimod) in clinical trials demonstrating that high efficacy DMTs do have improved efficacy over other DMTs in patients <40.5 years, but that DMTs generally provide no benefit >53 years (23). It is important to remember that this reflects treatment at the population level as we see that some older patients continue to have disease activity into their 60s or 70s. Our evaluation of an MS population in the real world suggests that high efficacy DMTs still have an additional benefit in patients that are older at least until they are 45 years in binomial models but until they are 54.2 years in linear models. This likely reflects the older population and lack of patients on lower efficacy platform DMTs when compared to the meta-analysis by Weideman et al. (23). Our study is a real-world study and is limited by our ability to control factors such as choosing between treatments and when/where to get MRIs. Therefore, age and disease activity are drivers of outcomes of DMT discontinuation. The Vienna-Innsbruck DMT discontinuation score predicts risk of disease reactivation after discontinuing glatiramer acetate or beta-interferons using a weighted calculation based on age, disease activity on MRI (≥3 new/enlarged T2 lesions or ≥1 gadolinium-enhancing lesion), and duration of stability (years since relapse or EDSS change) (24). In that study, patients age <45 years had a HR 4.3 (2.5, 7.1), *p* < 0.001 and patients age ≥45 and <55 years HR 2.1 (1.4, 3.8), *p* < 0.001, as compared to patients ≥55 years. In addition, activity on MRI <6 months prior to discontinuation had a HR 3.9 (3.2, 4.9), *p* < 0.001, and duration of stability <4 years had a HR 4.4 (2.7, 8.3), *p* < 0.001 and ≥4 and <8 years HR 2.3 (1.6, 4.5), *p* < 0.001, when compared to stability ≥8 years. Ultimately, identification of patients most likely to need ongoing DMT will benefit from multimodal predictive models encompassing radiological, clinical, and other biomarker data, in addition to age and other demographic factors.

### Variable Therapy Risks Over a Lifetime

In addition to concerns related to decreased therapeutic impact from DMT with aging and duration of disease, there are substantial concerns that aging increases risks of DMT use. The most pronounced concern is risk of severe infection and the direct relationship of this risk with aging and disability. Multiple sclerosis patients are at increased risk of infections, particularly infections of the respiratory tract and urinary tract (4). Multiple sclerosis patients are also at increased risk for hospitalization from infections (i.e., severe infections) when compared to patients with rheumatological conditions, likely underscoring the impact of accumulated disability from MS (25). In the context of accumulated disability from MS and overall reduced lymphocyte production because of immunosenescence, infection risk increases with age and immunosuppressive DMT (either through impaired trafficking, cellular depletion, or hypogammaglobulinemia depending on DMT mechanism).

Overall serious infections (resulting in hospitalization) in a nationwide Swedish cohort were higher for rituximab (HR 1.7 compared to beta-interferons or glatiramer acetate) than for other DMTs (26). This study found that age needed to be accounted for in these comparisons as infection risk increased with age, as well as disability, lymphopenia, hypogammaglobulinemia, and treatment duration. We evaluated these factors recently in multivariate models on 1,000 patients on long-term rituximab (9). We verified that all of these factors contributed to infections including male gender [OR 2.16 (1.24, 3.77)], rituximab treatment duration [OR 1.33 (1.17, 1.51)], and prior immunosuppression [OR 2.41 (1.19, 4.86)], but disability carried the most weight using stepwise selection models. However, disability had the largest effect of increasing the risk of serious infections [bilateral support (walker) OR 3.14 (1.34. 7.37); wheelchair OR 8.56 (4.47, 16.39)]. Hypogammaglobulinemia is a well-known adverse effect of anti-CD20 therapies and is associated with duration of use of B-cell depleting agents; low IgM may occur in up to 31% of patients and low IgG in up to 7% at 6 years (27).

Progressive multifocal leukoencephalopathy (PML) due to JC virus infection is a complication of treatment with multiple DMTs, most frequently natalizumab. In a 238-patient cohort study of natalizumab-treated patients who developed PML, age >50 years was associated with earlier onset (28). Further, age is associated with increased mortality from PML in the setting of natalizumab (5). Duration of natalizumab treatment is also a pronounced risk factor (29). Cases of PML, some with carry-over from natalizumab, have also been reported with other DMTs (notably with dimethyl fumarate and fingolimod) and may be more common in older patients (30–33). Other infectious complications are also related to aging and duration of treatment. Fingolimod-associated cryptococcal meningitis may be more frequent in older patients and those with treatment duration >2 years (34). Herpes zoster is also reported more frequently with older age across multiple DMTs (35). Concern regarding infections extends to the effects of COVID-19, for which there is additive risk for poor outcomes with anti-CD20 DMT (36, 37). The COVID-19 pandemic has also heightened the focus on how certain DMTs may reduce the effectiveness of COVID-19 vaccinations (38, 39). The VELOCE trial of ocrelizumab extends this concern to additional vaccines (40).

In addition, malignancy and other agent-specific adverse events are reported in some cases (6, 7). In a meta-regression of 45 trials utilizing DMTs with a variety of mechanisms, depletive DMTs (ocrelizumab and alemtuzumab) were associated with higher incidence of neoplasms, with an effect in those >45 years (41). While there was initial concern based on phase III trial data that ocrelizumab may lead to increased rates of breast cancer, this has not been demonstrated in an analysis of 11 clinical trials and post-marketing surveillance (27). Basal cell carcinoma, for which risk increases with age, has been reported with fingolimod (42, 43). Fingolimod-associated macular edema has also been more common in patients >41 years (44). Taken together, this suggest that non-infectious adverse events increase with age, but this data is not robust.

### De-escalation as a Treatment Strategy—Finding a Balance

The natural history of MS changes with aging, with less relapses and MRI disease activity. DMTs have little demonstrated benefit in progressive neurological dysfunction independent of relapses, especially in older patients. In addition, DMT safety may diminish with age, primarily due to increased risk of infections. Finally, most of the phase III trials resulting in approval of MS DMTs have been done in individuals <55 years, yet almost half of adults with MS are ≥55 years, meaning there is minimal safety and efficacy data in older patients with MS. Thus, whether it is necessary and safe to continue DMT as people age remains unclear. Observational studies suggest DMT may be able to be stopped safely later in life, but conditions and timing under which this may be done safely, with minimal risk of recurrent disease activity, remain unclear (45). To better understand these risks, two randomized, controlled, discontinuation trials are looking at discontinuing DMTs in older patients with MS. Discontinuation of DMTs in MS (DISCOMS, NCT03073603) is evaluating stopping DMTs in MS patients of all phenotypes who have been clinically stable for 5 years (radiologically stable for 3 years) and are ≥55 years. A second study looks at DMT Withdrawal in Inactive Secondary Progressive MS Patients Older Than 50 Years (STOP-I-SEP, NCT03653273) and requires clinical and radiological stability for 3 years. A third trial will examine discontinuing treatment in patients as young as 18 years but who have been stable clinically and radiologically for 5 years, Discontinuing DMT in Stable Relapsing-Onset MS (DOT-MS, NCT04260711).

Escalation, induction, and early high efficacy approaches may all be succeeded by ultimate DMT discontinuation over time, and risks of their use may not match with their potential benefits over the age spectrum of MS. As such, de-escalation (Figure 1F) is an extension of these emerging strategies that aims to match the potency of DMT with disease activity based on the natural history of MS and, aspirationally, biomarkers for disease activity. This may consist of extended interval or reduced dosing, or potentially transition to a less potent DMT.

De-escalations studies have often involved coming off natalizumab due to concerns with developing PML and include studies about switching to fingolimod and dimethyl fumarate (46, 47). These trials demonstrated that patients with shorter transition times did better and while there is a trend favoring conducting this transition in older patients (≥55 years) this was not adjusted for transition times (47). Though data for extended interval dosing and/or reduced dosing of high efficacy DMT is evolving, these strategies may result in preserved efficacy and reduced risk of adverse effects, in addition to economic advantages. Ultimately, de-escalation strategies according to the data presented above, appear to best match disease activity and deserve to be studied in better detail using randomized controlled trials optimized by use of personalized biomarkers for disease activity, as well as clinical and radiographic monitoring for relapse and disability.

Therefore, we propose a de-escalation treatment approach for patients with MS. High efficacy DMTs are disproportionately more efficacious early in the disease course arguing for early use. This also takes advantage of the relatively low concern for adverse events as these patients are typically younger and have low levels of disability. For these reasons, the benefit of high efficacy DMT is front-loaded. Due to decreasing DMT efficacy and increasing risks, as patients approach 40–55 years, de-escalating should be contemplated. In addition to age, disability (especially in patients who require bilateral support or are wheelchair-bound) should be considered, as should DMT-specific factors that increase infection risk such as JCV seroconversion for natalizumab, lymphocytes <500/μl for dimethyl fumarate, or hypogammaglobulinemia for B-cell depleting therapies. If the patient is clinically stable following de-escalation, discontinuation of DMT may then be an option. This process should be discussed with patients and adjusted based on comfort level and desire for aggressive treatment.

## Data Availability Statement

The raw data supporting the conclusions of this article will be made available by the authors, without undue reservation.

## Ethics Statement

The studies involving human participants were reviewed and approved by Colorado Multiple Institution Review Board (COMIRB) affiliated with the University of Colorado. Written informed consent for participation was not required for this study in accordance with the national legislation and the institutional requirements.

## Author Contributions

All authors provided substantial contributions to the conception and design of this work, drafting and revising the manuscript critically for important intellectual content, provided approval for publication of the content, and agreed to be accountable for all aspects of the work in ensuring that questions related to the accuracy or integrity of any part of the work are appropriately investigated and resolved.

## Conflict of Interest

JC has received research funding from MedDay, Novartis, EMD Serono, National Multiple Sclerosis Society, NIH, Immune Tolerance Network, Patient-Centered Outcomes Research Initiative. He has been editor of Neurology: Clinical Practice and is currently the associate editor of Annals of Neurology. EA has received compensation for activities such as advisory boards, lectures, and consultancy with the following companies and organizations: Actelion/Janssen, Alexion, Bayer, Biogen, Celgene/BMS, EMD Serono/Merck, Genentech/Roche, Novartis, Sanofi, and TG Therapeutics and research support from: Biogen, Genentech/Roche, Novartis, TG Therapeutics, Patient-Centered Outcomes Research Initiative, National Multiple Sclerosis Society, National Institutes of Health, and Rocky Mountain MS Center. The remaining authors declare that the research was conducted in the absence of any commercial or financial relationships that could be construed as a potential conflict of interest.

## Publisher's Note

All claims expressed in this article are solely those of the authors and do not necessarily represent those of their affiliated organizations, or those of the publisher, the editors and the reviewers. Any product that may be evaluated in this article, or claim that may be made by its manufacturer, is not guaranteed or endorsed by the publisher.
