# Supplementary material for: Evolution of Disease Modifying Therapy Benefits and Risks: An Argument for De-escalation as a Treatment Paradigm for Patients With Multiple Sclerosis
Source: Front Neurol. 2022 Jan 25;12:799138. doi: 10.3389/fneur.2021.799138 (PMC8821102; doi:10.3389/fneur.2021.799138)
Supplement: Supplementary file 1 [file Table_1.DOCX]

**Supplemental Table 1.** Adjusted and unadjusted odds ratios comparing the effect of oral vs infusible medications by patient age.

|  | **< 45 Years of Age** | | | **≥ 45 Years of Age** | | |
| --- | --- | --- | --- | --- | --- | --- |
|  | **N** | **Odds Ratio**  **(95% CI)** | **p-value** | **N** | **Odds Ratio**  **(95% CI)** | **p-value** |
| **Simple Logistic Regression** | 625 | 2.67  (1.89, 3.75) | <0.001 | 379 | 1.60  (0.96, 2.64) | 0.069 |
| **Adjusted Logistic Regression*** | 625 | 2.89  (2.02, 4.13) | <0.001 | 379 | 1.65  (0.99, 2.76) | 0.053 |
| **Propensity Matching with 1:1 nearest neighbor matching with replacement*, with covariates** | 552  (440 unique) | 2.18  (1.34, 3.53) | 0.002 | 446  (333 unique) | 1.16  (0.59, 2.27) | 0.675 |
| *controlling for age, disease duration, gender, contrast enhancement on baseline MRI, and base disease burden | | | | | | |
